# Caffeine and Bones: If Less Is Good, More May Not Be Better

**DOI:** 10.1089/caff.2019.29011.ah

**Published:** 2019-06-19

**Authors:** Luca Antonioli, György Haskó

**Affiliations:** ^1^ Department of Clinical and Experimental Medicine, University of Pisa, Pisa, Italy.; ^2^ Department of Anesthesiology, Columbia University, New York, New York.

1,3,7-Trimethylxanthine, popularly known as caffeine, is a pharmacologically active constituent contained in several foods, beverages, dietary supplements, and drugs.^1^ The main natural contributor of caffeine to the diet is coffee, which is one of the most widely consumed beverages worldwide.^1^

Over the years, caffeine has been shown to have myriad effects on the human body, garnering both favorable and unfavorable attention from the scientific community, the press, and the Non-Government Organizations. In particular, the main question is: “Is caffeine safe and how much could be too much?”.^1^

Several lines of preclinical and clinical evidence demonstrated that consuming coffee, owing to its various ingredients, which include caffeine as well as antioxidants and other bioactive compounds, may help protect the human brain and, therefore, lower the risk of developing some neurodegenerative diseases (e.g., Alzheimer's and Parkinson's). In addition, other studies have shown that coffee may reduce the risk of metabolic disorders (diabetes, gallstones, and liver cirrhosis), and as these beneficial metabolic effects of coffee appear to be inversely associated with the serum levels of inflammatory biomarkers, coffee may potentially exert its protective effects by decreasing inflammation.^2,3^ A number of recent studies have provided hints that these protective effects of coffee are mediated by the anti-inflammatory and antioxidant properties of caffeine.^4,5^ However, there is no clear consensus on the mechanisms whereby caffeine and other xanthines influence inflammation. Caffeine causes most of its biological effects through antagonizing all types of adenosine receptors, which are the A_1_, A_2A_, A_2B_, and A_3_ receptors. As adenosine receptor activation actually curbs the inflammatory process by suppressing proinflammatory mediators (i.e., TNF, IL-12, and nitric oxide) and increasing anti-inflammatory cytokine production (i.e., IL-10),^6,7^ it is hard to see how caffeine could exert its anti-inflammatory effect through blockade of adenosine receptors.

In contrast, other studies have indicated coffee intake as a modifiable risk factor for poor health. In particular, since coffee contains caffeine, a stimulant, coffee drinking is not generally considered to be part of a healthy lifestyle, at least when consumed in excess. In this regard, health and regulatory authorities have highlighted the risk of caffeine consumption among specific populations, such as pregnant and lactating women, children, adolescents, young adults, and people with underlying heart and other health conditions.^8^

Indeed, excessive consumption of caffeine can cause insomnia, nervousness, restlessness, irritability, upset digestive tract, fast heartbeat, and even muscle tremors. In addition, owing to its diuretic effects, caffeine can cause dehydration and compromised fluid balance.^9^ Of note, such electrolyte alterations induced by the consumption of caffeine have been associated with a reduction of bone mass by inducing urinary calcium loss and decreasing bone mineral density.^10^

In light of these concerns, a number of epidemiological studies have been performed to investigate the effect of coffee on bone metabolism, which have had mixed results. While some studies revealed a link occurring between the consumption of caffeine and an increased risk of fractures in women, this evidence has been contested by others.^11^

In parallel, a series of preclinical studies have focused their attention on exploring how and to what extent caffeine affects osteoclast and osteoblast differentiation and function. In this context, Choi et al. demonstrated that caffeine enhances osteoclastogenesis, suggesting an involvement of caffeine in osteoclast-associated diseases including osteoporosis.^12^ In addition, *in vitro* studies have also revealed direct or indirect deleterious effects of caffeine on osteoblasts.^13^ In particular, the study by Fernandes et al., published in this issue of *Journal of Caffeine and Adenosine Research*, focused its attention on the *in vitro* effect of exposure to low or moderate caffeine concentrations on osteoblastic cells isolated from ovariectomized rat. The authors provided evidence that low caffeine concentrations maintained functional activity and the expression of osteogenetic genes (a downregulation of *Alp* and *Bsp* as well as a downregulation of *Runx2* and *Bglap*) in osteoblastic cells obtained from osteoporotic rats. By contrast, moderate caffeine concentrations delayed the expression of osteogenetic genes, thereby disturbing functional activity and consequently extracellular bone mineralization. Of note, the authors proposed that the beneficial effects of low caffeine concentrations on the expression of genes may be ascribed caffeine's antioxidant capacity through scavenging •OH species.

Overall, the trend that emerges from the various clinical studies is that moderate caffeine intake is generally considered to exert no or even positive effects on human health, with some caveats. That is, despite the observations that daily caffeine intake of up to 400 mg/day for the healthy adult population is not associated with adverse cardiovascular health, behavior, cancer risk, male fertility, calcium homeostasis, and bone health,^14^ the nonuniformity of caffeine sources in the various studies makes the interpretation of results difficult, since most caffeinated products contain other compounds that can influence disease risk.

Indeed, assessing the effects of caffeine in humans is very challenging, in part, because of differences in the caffeine content of beverages and food products and in part because of the variations in metabolism of caffeine among individuals. In research settings, the number of cups of coffee per day is frequently used as a surrogate marker for caffeine exposure. However, this marker may not represent caffeine exposure accurately, since caffeine intake varies significantly by type of coffee (espresso, instant coffee, and brewed coffee), by manufacturing method, as well as from other beverages (such as tea or energy drinks) and food products. In parallel, another point worth considering is the high variability among people in caffeine metabolism. Both clinical and epidemiologic studies have identified genetic variants able to affect caffeine metabolism as well as coffee consumption,^15^ thus making difficult to accurately evaluate the dose that can have detrimental effects.

In conclusion, based on the available evidence, it emerges that moderate consumption of caffeine appears to exert a positive effect on the cardiovascular and central nervous systems, and several metabolic indexes, which may include bone metabolism. However, a valid method for assessing caffeine exposure is mandatory before results confirming caffeine's effects on bone health can be obtained.
